# Supplementary figures and images for: In vivo Polycystin-1 interactome using a novel Pkd1 knock-in mouse model
Source: PLoS One. 2023 Aug 4;18(8):e0289778. doi: 10.1371/journal.pone.0289778 (PMC10403143; doi:10.1371/journal.pone.0289778)

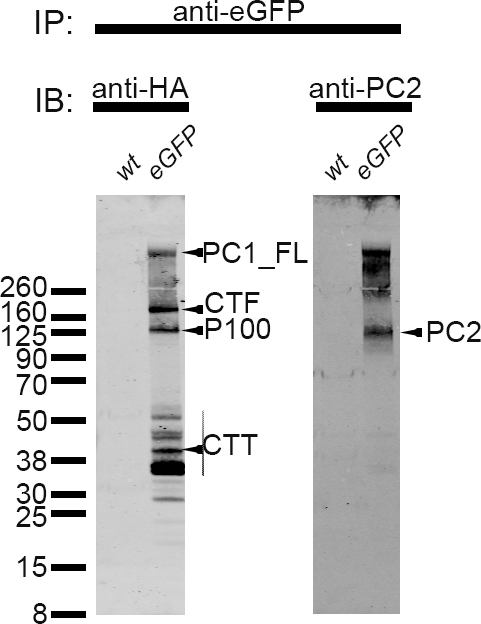

Supplement: S1 Fig — The left panel PC1 shows full-length (PC1_FL), cleavage products (PC1_CT; P100), and multiple CTT-related cleavage products (presumed CTT marked with an arrow; dotted line represents range of possible CTT products). The right panel is the same blot, probed with anti-PC2 and detected in a different wavelength, showing PC2 co-immunoprecipitation. PC2 appears to occasionally form oligomers depending on denaturing conditions (unpublished previous observations and 34). (JPG) [file pone.0289778.s001.jpg]

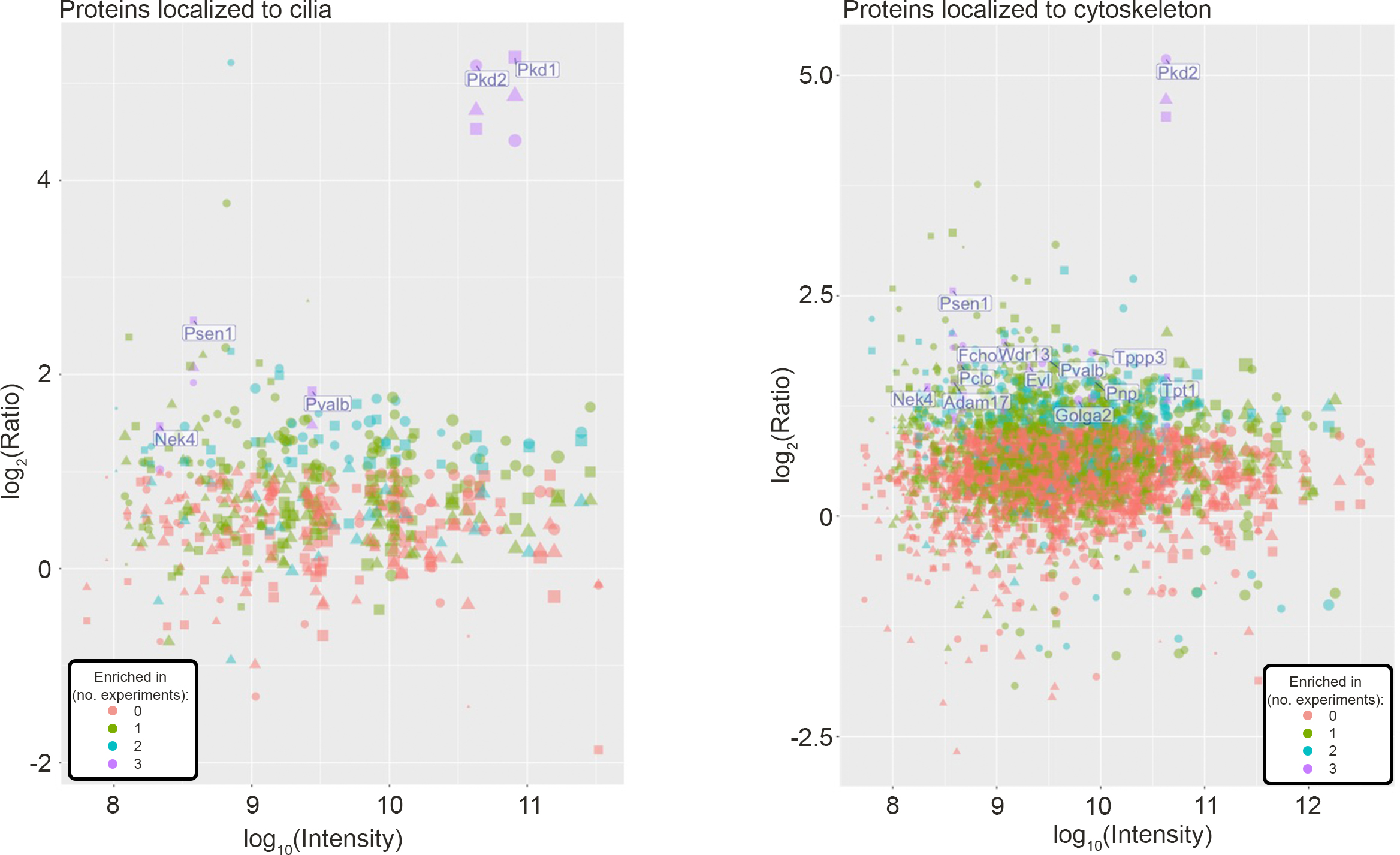

Supplement: S2 Fig — The axes show the log10(intensity) and log2(intensity ratio between knock-in and control samples). Each dot corresponds to one detected protein; shape (circle, square, triangle) represents experimental batch; the colors summarize the number of times the enrichment ratio was above 2 for the protein: 0-orange/red; 1-green; 2-blue;3-purple. Text identifies proteins that had enrichment ratios >2 in each of the three experiments. The panels show proteins reported in cilia (left) or cytoskeleton (right). (JPG) [file pone.0289778.s002.jpg]

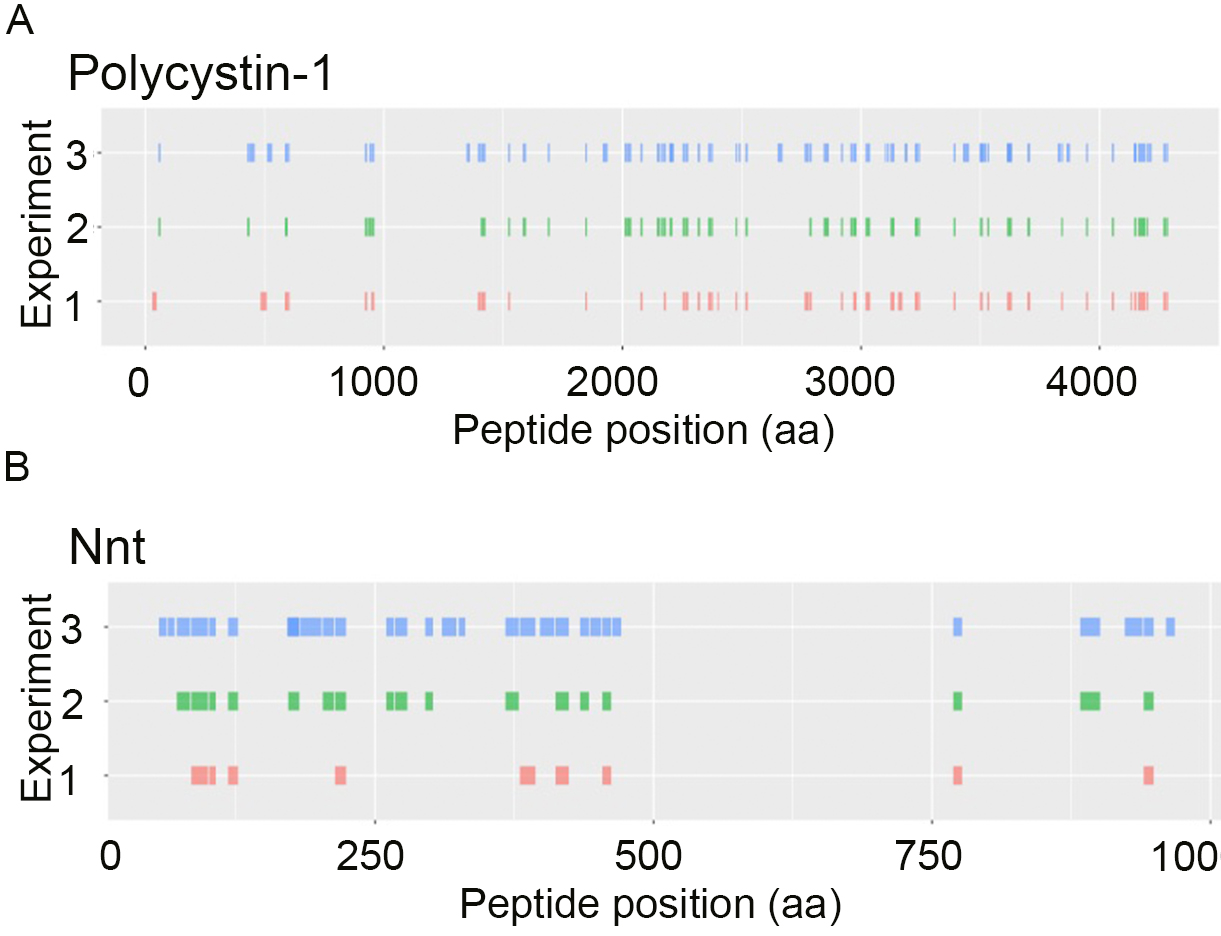

Supplement: S3 Fig — The data for each of the three experiments are shown. eGFP was also identified in each of the three experiments but not shown here or included in S1 Table since it is not part of the native protein nor is it an independent interactor of PC1. (JPG) [file pone.0289778.s003.jpg]

Fig1 G

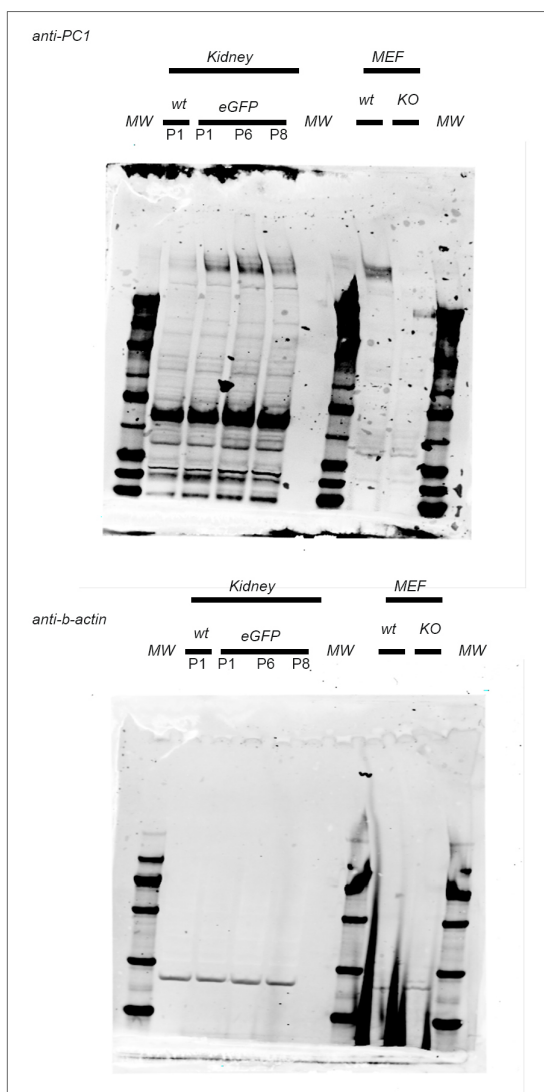

Fig1 H

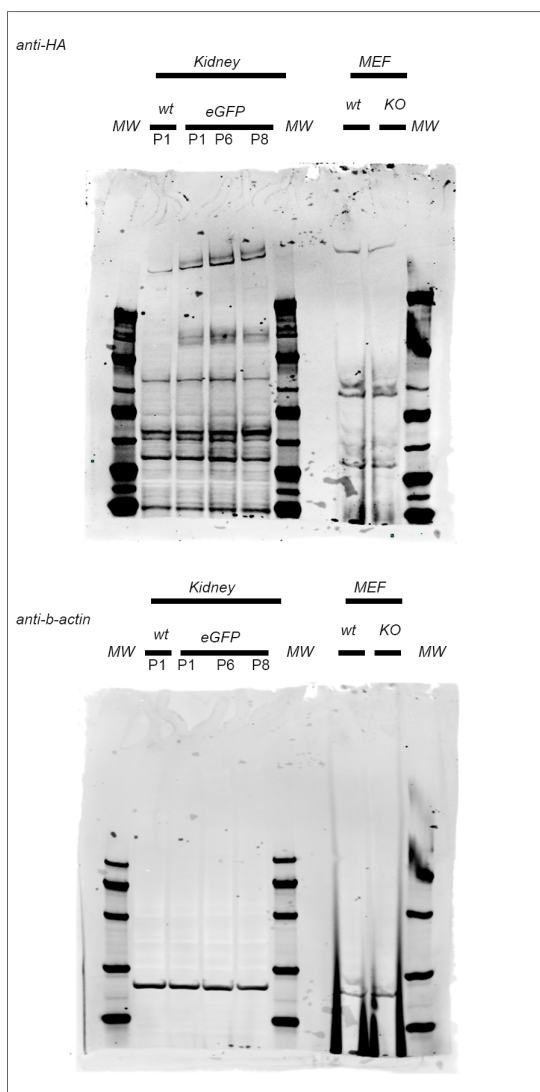

Fig1 I

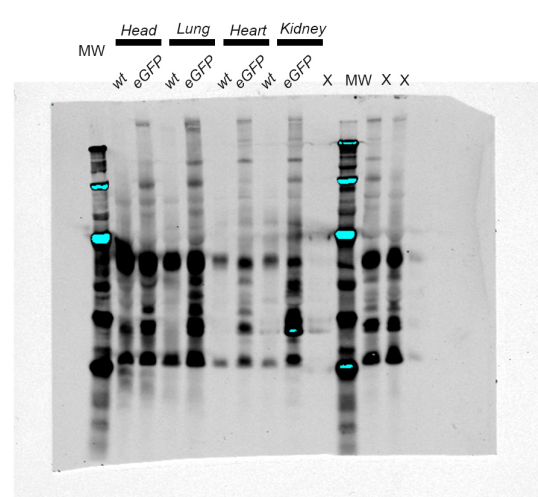

Fig1 J

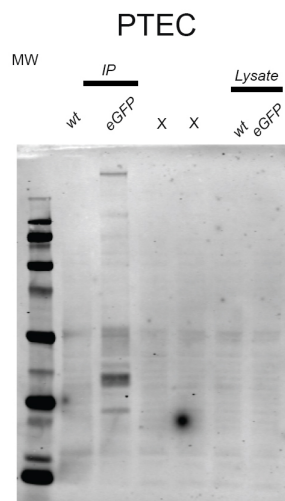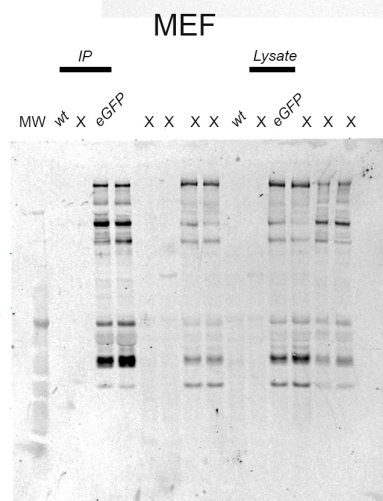

All images were captured using the Li-Cor Odyssey Infrared Imaging System

Supplement: S1 Raw images — This figure also includes in Fig 1G (on left) uncropped images of protein lysates isolated from Murine Embryonic Fibroblasts (MEFS) and probed with 7e12 antibody to test for the monoclonal’s specificity. “WT” indicates lysate from a normal control mouse while”KO” identifies lysates of MEFs from a Pkd1 null mouse [25]. PC1 is only detected in the control sample. The right panel is of lysates from the same samples shown on the left probed with an anti-HA monoclonal. Different blots were used for the two panels because both primary antibodies are of mouse origin. Anti-HA detects multiple non-specific bands in both kidney and MEFs. (PDF) [file pone.0289778.s004.pdf]
